# Pathology

**Published:** 1859-01

**Authors:** 


### Foreign Abstracts,

CHIEFLY FRENCH, GERMAN, AND ITALIAN; OR, BI-MONTHLY NOVELTIES IN MEDICAL, SURGICAL, OBSTETRICAL, AND CHEMICAL SCIENCE. BY EMIL FISCHER, M.D.

### PATHOLOGY.

I.—*On Ulceration and Perforation of the Diaphragm in Peritonitis.* By Dr. E. Bonamy.

A man, twenty-seven years of age, was admitted into the Hospital of Nantes for a spontaneous, general peritonitis, which was accompanied by the ordinary symptoms. On the nineteenth day an abscess opened at the umbilicus. On the twenty-second, acute pain was felt on the right side of the thorax. The next day was present frequent cough, expectoration of purulent sputa, more fetid than the liquid which passed through the umbilicus, and having an analogous odor; in the inferior third of the thorax, on the right side and posteriorly there was deep and distinct amphoric sound, which appeared to proceed from the depth of the abdomen; at the same time complete abatement of the abdominal symptoms; abdomen painless and distended, and the discharge of purulent serum through the umbilicus ceased; face contracted. From this moment the state of the patient became daily worse, and death supervened on the twenty-eighth day of the disease. On the previous day it had been observed, that, with each respiration, a loud gurgling in the abdomen was produced, and that bubbles of air escaped through the umbilicus.

On post-mortem examination, the usual characteristics of peritonitis, with pseudo-membranes, etc., were found; the right pleura communicated with the abdominal cavity by a canal, which was formed on one side by the thoracic parietes, on the other by the liver deprived of its capsule, and completed by false membranes; this canal terminated in the pleura by a large opening of the diaphragm; the right lung receded to a level with the seventh rib, and communicated by a broncho-pleural fistula with the purulent collection of the pleura; all the bronchi were filled with pus; the left lung adhered to the diaphragm, which was much reduced in thickness; at the seventh rib, and underneath it, the purulent collection existed, perfectly circumscribed by pseudo-membranous adhesions, and situated between the spleen, the great cul-de-sac of the stomach, and the left extremity of the liver.

M. Bonamy remarks, with reference to this fact, that the inflammation of the tissues, to which the inflamed peritoneum adheres, tends, sometimes, to bring about softening, atrophy, and destruction of these tissues. This result, which a certain chemical composition of the effused liquid contributes, perhaps, to produce, is advantageous, if it effects the escape of the effusion through the umbilicus; and in such a case, this discharge can, in a certain measure, be facilitated by means of dry-cups.

M. Malherbe saw, also, an atrophy of the diaphragm produced under the influence of a peritonitis, as in the patient of M. Bonamy. M. Scoutetten (*Archives de Médecine*, vol. i. 1824,) reported a case, in which the diaphragm was perforated by an ulcerative process, which, however, progressed in an opposite direction from the pleura to the peritoneum. (*Journal de la Société Académique de la Loire Inférieure*, tome xxiv. No. 177.)

